# Pathways through which health influences early retirement: a qualitative study

**DOI:** 10.1186/1471-2458-13-292

**Published:** 2013-04-03

**Authors:** Astrid de Wind, Goedele A Geuskens, Kerstin G Reeuwijk, Marjan J Westerman, Jan Fekke Ybema, Alex Burdorf, Paulien M Bongers, Allard J van der Beek

**Affiliations:** 1 Department of Public and Occupational Health, the EMGO+ Institute for Health and Care Research, VU University Medical Center, Amsterdam, The Netherlands; 2 Department of Work, Health & Care, Netherlands Organisation for Applied Scientific Research TNO, Hoofddorp, The Netherlands; 3 Body@Work, Research Center on Physical Activity, Work and Health, TNO-VU/VUmc, Amsterdam, The Netherlands; 4 Department of Methodology and Statistics, Institute of Health Sciences and the EMGO+ Institute for Health and Care Research, VU University, Amsterdam, The Netherlands; 5 Department of Public Health, Erasmus MC, University Medical Center Rotterdam, Rotterdam, The Netherlands

**Keywords:** Early retirement, Health, Qualitative study, Dialogue

## Abstract

**Background:**

Due to the aeging of the population, there is a societal need for workers to prolong their working lives. In the Netherlands, many employees still leave the workforce before the official retirement age of 65. Previous quantitative research showed that poor self-perceived health is a risk factor of (non-disability) early retirement. However, little is known on how poor health may lead to early retirement, and why poor health leads to early retirement in some employees, but not in others. Therefore, the present qualitative study aims to identify in which ways health influences early retirement.

**Methods:**

Face-to-face semi-structured interviews were conducted with 30 employees (60–64 years) who retired before the official retirement age of 65. Participants were selected from the Study on Transitions in Employment, Ability and Motivation. The interviews were transcribed verbatim, a summary was made including a timeline, and the interviews were open coded.

**Results:**

In 15 of the 30 persons, health played a role in early retirement. Both poor and good health influenced early retirement. For poor health, four pathways were identified. First, employees felt unable to work at all due to health problems. Second, health problems resulted in a self-perceived (future) decline in the ability to work, and employees chose to retire early. Third, employees with health problems were afraid of a further decline in health, and chose to retire early. Fourth, employees with poor health retired early because they felt pushed out by their employer, although they themselves did not experience a reduced work ability. A good health influenced early retirement, since persons wanted to enjoy life while their health still allowed to do so. The financial opportunity to retire sometimes triggered the influence of poor health on early retirement, and often triggered the influence of good health. Employees and employers barely discussed opportunities to prolong working life.

**Conclusions:**

Poor and good health influence early retirement via several different pathways. To prolong working life, a dialogue between employers and employees and tailored work-related interventions may be helpful.

## Background

Many industrialized countries are confronted with an aeging workforce, since the baby-boom generation ages, fertility rates have declined and younger workers enter the labour market later [1]. The ratio of retired elderly to the active working population is increasing, which causes a higher pressure on public finance. Therefore, there is a societal need for workers to extend their working lives. Although the average retirement age in the Netherlands increased from 60.9 years in 2001 to 63.1 years in 2011, many workers still leave the workforce before the official retirement age of 65 [2].

Previous research on work disability pensions showed that workers with specific diseases, such as depression [3], rheumatoid arthritis [4], diabetes [5], or cancer [6] have a higher risk of an early exit from the work force due to work disability. A recent review study showed that poor perceived health is also a risk factor of early retirement without compensated work disability [7], though the association is less strong than with disability pensions [8]. In this review moderately increased risks of early retirement were found in four of the six studies that were included (OR/HR/RR 1.28 to 1.86), a high risk was found in one study (OR 3.36) and no significant relation between health and early retirement was found in another study. The differences between these studies might be explained by the fact that poor perceived health may result in early retirement in some employees or circumstances, but not in others.

Although several longitudinal studies identified poor health as a predictor of early retirement, there is little understanding on *how* poor health may lead to early retirement. Second, little is known on *why* poor health leads to early retirement in some situations, but not in others. Third, the influence of good health on early retirement has barely been studied. More insight in the role of health in early retirement could be helpful to design interventions aiming to prolong persons working life despite health problems. Hence, the present study aims to identify through which pathways health influences early retirement.

## Methods

### Design

This qualitative study was part of a larger qualitative investigation on why persons retire early. The present study focuses on health-related reasons of early retirement. Non-health related reasons of early retirement are described elsewhere (Reeuwijk, De Wind, Westerman, Ybema, Van der Beek, Geuskens, submitted).

Face-to-face semi-structured interviews with Dutch employees who retired early were conducted. Early retirement referred to retirement before the official retirement age of 65, and excluded persons who retired early due to (partially) compensated work disability or unemployment. Persons reporting compensated work disability or unemployment were excluded because previous research suggested that different factors underlie these transitions out of work [9].

### Study participants

The participants were selected from the Study on Transitions in Employment, Ability and Motivation (STREAM) [10]. The aim of this prospective cohort study is to identify in what circumstances persons aged 45 to 64 years prolong their working life, while maintaining good health and good work productivity. In total 15,118 persons were included in STREAM in 2010.

Inclusion criteria for the present study were: (1) persons had a paid job as an employee at the time of STREAM 2010, (2) retired before the age of 65 in the last 12 months or were going to retire early in the next six months and already formally arranged this with their employer at the time of the interview, and (3) were aged 58 to 64 years at the time of the interview. Moreover persons had given permission in the STREAM 2010 questionnaire to be contacted for additional research.

To ensure heterogeneity in the study participants, participants were purposefully selected [11] based on age, educational level, and their intention to retire in 2010. We selected on age, since different reasons might underlie retirement in those that retired at a relatively young age (e.g. 59 years) compared to those that retired at a higher age (e.g. 64 years). Similarly, educational differences in reasons of early retirement may exist, e.g. due to exposure to different physical and psychosocial working conditions. The intention to retire was assessed with one question in the STREAM questionnaire in 2010, i.e. ‘Are you planning to stop working in the next 12 months?’. This item could be answered on a 5-point Likert scale ranging from ‘certainly not’ to ‘certainly’. Persons who answered ‘maybe’, ‘probably’ or ‘certainly’ were eligible to be contacted for the present study. We selected purposefully on this characteristic to assure that both persons in which longstanding processes and persons in which more sudden events influenced early retirement were included. We chose not to contact persons who answered ‘certainly not’ or ‘probably not’, because we assumed early retirement would be rare in these persons. Health was not taken into account in the selection of participants.

**Figure 1:**
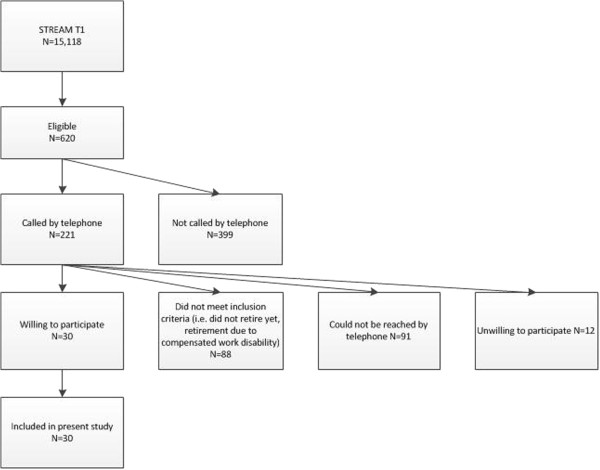
Inclusion of the study participants.

Between July 2011 and October 2011, 221 persons were contacted by telephone to check whether they met the inclusion criteria (Figure 1). The aim and content of the interview study was explained and their willingness to participate in a face-to-face interview was checked. Eighty-eight persons did not meet the selection criteria. They either had not retired yet and no formalized arrangements with their employer to do so in the next six months, or went on early retirement due to compensated work disability. In total 91 persons could not be reached by telephone. These persons were called at least once again after one or two weeks. Since telephone calls were made during day time on working days, it could be hypothesized that a substantial proportion of those that could not be reached were still employed. Twelve persons were unwilling to participate. Reasons were personal circumstances (N=4), no time (N=2), unwillingness to talk about work history and early retirement (N=2), and miscellaneous reasons (N=4). The first 30 persons who were eligible and gave permission for an interview were enrolled in this study.

### Interview protocol

Prior to the beginning of the study, a comprehensive semi-structured interview guide was created based on the life course perspective [12] and determinants of early retirement according to the literature. The life course perspective considers transitions from work to retirement as a part of the life course. The processes leading to the transition are influenced by someone’s individual history and characteristics, and the context of the transition. The life course perspective has previously been used to understand how persons experience (the transition to) retirement [12]. According to the literature, early retirement may be influenced by determinants in the following domains: health, job characteristics, skills and knowledge, social factors, and financial factors [7,13-16]. The interview guide was pilot tested, and minor adjustments were made.

Before the start of the interview, the interviewer introduced herself, and again explained the aim and content of the study and the interview. Subsequently, open-ended questions focused on six topics. The first part was aimed at getting acquainted with the interviewee and focused on the personal situation and home situation. The second part was directed to the person’s work history and job-job transitions. Together with the participant, the interviewer created a timeline of the interviewee’s work history and other important (positive or negative) events, such as education, marriage, divorce, birth, death of family or friends, and periods of illness. The third part focused on the reasons why an interviewee had retired early, or had made arrangements to do so. Understanding of the reasons for early retirement was gained through in-depth follow-up questions. For example, if a person mentioned poor health as a reason for early retirement, the interviewer asked *why* poor health was a reason for early retirement, *since when* poor health was a problem, and *how* poor health had caused early retirement. The fourth part focused on the timing of the transition from work to early retirement. The fifth part focused on circumstances under which the interviewee would have prolonged working life. The last part of the interview concentrated on satisfaction with the transition from work to early retirement, the present situation, and the perceived future.

### Interview procedure

The interviews were carried out by the first author (AdW). The interviewer was familiar with interview techniques, such as clarification, paraphrasing and summarizing. During most of the interviews, a second interviewer was present who took notes (KR or DR). The interviewers did not have a prior relationship with any of the participants. The interviews were carried out in participants’ homes throughout The Netherlands, except for one person, who, upon request, was interviewed at work. Interviews were digitally recorded. All participants agreed to this procedure. On average interviews lasted 80 min (range: 40–156 min). During 9 interviews non-participants were present (spouse (N=7), spouse and daughter (N=1), and granddaughter (N=1)). In one interview the spouse helped the respondent come up with ideas about what was asked. In two interviews the spouse interfered substantially. Issues brought up by these spouses were interpreted with caution in the analysis.

### Analysis

Analysis of the interviews took place in four steps. First, the interviews were transcribed fully verbatim. All interviews were listened to at least twice and compared to the transcriptions to check accuracy.

Second, 10 interviews were independently summarized, using transcriptions and field notes, and open-coded by AdW and KR. The aim of this step was to understand why and how the transition from work to early retirement had taken place in these persons. Afterwards, AdW and KR discussed summaries, timelines, codes, and coding trees extensively, and reached consensus.

In the third step, the remaining 20 interviews were summarized, and open-coded by either AdW or KR. Summaries and coded interviews were cross-checked, and AdW and KR regularly met to discuss findings. During these meetings, data saturation was monitored. No new information on reasons of early retirement was derived from the last interviews.

In the fourth step, AdW extracted parts about health from the transcriptions of all interviews. AdW open coded these parts in more detail, and discussed the findings extensively with KR. The aim of this step was to investigate the role of health in the transition from work to early retirement in more detail.

Parallel to the four steps described above, AdW and KR regularly met to compare interviews. Leading questions during these discussions were: (1) what similarities can be identified between the stories of the interviewees, and (2) why did certain processes take place in some persons, but not in others. To enhance robustness of the findings, main results were also discussed with other project members (MW and GG). In order to manage the data of the interviews, the computer package for qualitative analysis Atlas.ti 6 was used.

### Ethical considerations

The Medical Ethics Committee of the VU University Medical Center Amsterdam declared that no ethical approval was needed to conduct this study. Informed consent was obtained verbally from all participants during the telephone conversation, in which persons were invited for the interview.

## Results

Characteristics of the study participants are shown in Table 1. Twenty-three persons retired in the last 12 months and 7 employees were going to retire in the coming six months. The median of the retirement age was 61 years (range 60–64). In total 15 of the 30 participants mentioned that their own health played a role in early retirement. Health influenced early retirement in various ways. First, physical and mental health problems influenced early retirement. Second, ‘good health’ emerged as a factor that influenced early retirement.

**Table 1:** Characteristics of the study participants

**Interview**	**Gender**	**Age (median=62)**	**Retirement age (median=61)**	**Occupation**	**Educational level**	**Health played a role in early retirement (yes / no)**
1	Male	61	60	Solution manager IT	High	Yes
2	Male	60	60	Mechanic and chauffeur	Low	Yes
3	Male	60	60	Policeman	Low	Yes
4	Male	62	62	Teacher secondary school - history	High	Yes
5	Female	62	61	Administrative assistant	Low	No
6	Female	61	61	Physiotherapist	High	No
7	Male	61	61	Database administrator	High	No
8	Male	61	60	Service employee in public transport	Low	Yes
9	Male	60	60	Sales engineer	Intermediate	Yes
10	Male	62	62	Security officer	Intermediate	Yes
11	Male	62	61	Stockpile manager	Low	No
12	Male	62	62	Teacher secondary school - economics	High	Yes
13	Male	63	63	Civil servant	High	No
14	Male	62	61	Graphical designer	Intermediate	No
15	Male	63	64	Civil servant	Low	No
16	Female	61	61	Teacher primary school	High	Yes
17	Male	64	63	Teacher secondary school - mathematics	High	No
18	Male	61	60	Financial controller	High	No
19	Male	62	62	Consultant IT	Low	No
20	Male	62	61	Employee personnel department and administrative assistant	High	Yes
21	Male	61	61	Trouble shooter in machine construction	Low	No
22	Male	64	64	Financial controller	High	No
23	Female	63	62	Employee personnel department	Intermediate	Yes
24	Male	62	62	Teacher secondary school - mathematics	High	Yes
25	Male	60	60	Service technician	Low	No
26	Male	63	62	Job coach	High	Yes
27	Male	60	60	Civil servant	Low	Yes
28	Female	64	63	Administrative assistant	Low	No
29	Male	61	60	Mechanical chipper	Low	No
30	Female	60	60	Nurse	High	Yes

### Poor health

‘Poor health’ was (one of) the reason(s) to retire early in 12 of the 30 participants. Most persons who mainly retired early because of ‘poor health’ experienced a gradual decline in health. Poor health was the main reason to retire in some persons, whereas it was one of a variety of reasons in others. Poor health resulted in early retirement through four different pathways.

First, poor health resulted in early retirement in one employee who felt unable to work at all due to health problems, and felt there was no other possibility but to retire early. This 63-year old woman who had suffered from psychological disorders (ADHD and burnout) for many years had quitted paid employment after being granted a work disability pension four years ago. She explained:

“In 2007, that was the last year, I became sick in June or July. I worked till autumn, although I said ‘It’s not possible, I am too tired’. The company doctor said ‘No you have to’. I said ‘I can’t do that’, however, I started again. In March I completely collapsed. And in May the general practitioner concluded that I was depressed.”

However, during re-examination of her work disability pension, her insurance physician approved her for a 16 hour working week. Both this woman and her employer thought she was not able to work anymore, and together they concluded early retirement would be the best solution. She experienced early retirement as the only possible escape from work.

Second, persons who experienced or expected that physical or mental health problems caused a decline in their (future) ability to work decided to retire early (N=5). Health problems made their work physically or mentally (too) demanding, which resulted in feelings of incapability to accomplish working tasks properly. A 63-year old male job coach in a social workplace with a history of heart disease and cancer explained that he retired early because of health problems. He stated that work had been very important to him. Besides, it offered day structure and social contacts. This man felt he was not able to accomplish working tasks properly and explained:

“I was there to pump people up, to make them feel good, and now I got the feeling that I needed to be pumped up… and I thought that’s not good, because then you can’t help people in the way that you want to anymore.”

This man also felt that he was a burden to his colleagues by being absent on a regularly basis.

“The most important reason for me to stop was that I was sick…the work that you miss burdens your colleagues, because they have to take over.”

In some persons, negative events at work or certain working conditions, such as aggression, high work pressure, and conflicts, underlied the influence of poor health on early retirement. For example, a 60-year old policeman explained that a traumatic experience when he just started working had bothered him during the rest of his career. He explained that he had suffered from increasing fears in stressful situations and a declining mental health. This was the main reason for him to retire as soon as possible.

“I worked for the police for a year and a half, and then in a fight at a pub I got beat up so bad I had to go to the hospital. Well, I probably didn’t deal with that very well, because six weeks later I was working again. At that time they didn’t have a company support group, which luckily they do have now… I really experienced that as a burden during my whole career.”

He reported that he did not always perform well due to his traumatic experience:

“Just that in a violent situation, that could escalate I hid behind my colleague, or because of the violence I would intervene too quickly.”

Third, poor health influenced early retirement in persons with health problems who were afraid of a further decline in health (N=4). For example, a 60-year old civil servant who had suffered from many health problems during his life (e.g. Crohn’s disease, Transient Ischaemic Attack) was afraid to become disabled if he would continue working:

“Look, deciding to stop at 60… that was actually because I didn’t want to end up handicapped early.” […] “When I got that TIA and the oppression complaints, I said ‘60 is my limit’. I don’t want to think of being 62 or 63 and it going down hill from then on, and I get disabled. Then I'd have no one to blame but myself.”

Although this man experienced health problems, these problems did not influence his ability to work:

“Yes, but it didn’t limit me in my work. I had problems with walking to my work, or when I needed to walk up a lot of stairs. However, in my work it didn’t limit me at all.”

Similarly, a 60-year old woman, who worked as a nurse, explained that fear of a further decline in health played a role in her early retirement. She said that she valued her (future) health more than her work:

“Because my back is more important than work. That’s how I think about that. I don’t want to end up in a wheelchair in two years. So yeah, these things together made me retire.”

In the second and third pathway discussed above, the decision to retire early was often triggered by the financial opportunity to do so. Participants either had personal savings, or could claim a special financial arrangement from their employers. The 60-year old civil servant, mentioned above, for example, explained it would be stupid to risk his health by continuing to work.

“And that while I can get a nice arrangement from the age of 60 onwards.”

No pressure was felt from the employer or colleagues to retire early. Most persons did not discuss opportunities to (further) adjust the demands of the job to their abilities with their employer. One person reported that she proposed job redesign to extend working life, but according to her employer there were no opportunities within the organisation to do so. Although early retirement was experienced to be voluntary, most of these persons originally had not intended to retire (this) early.

Fourth, some employees felt capable of doing their job despite their health problems. Nevertheless, the employer suggested to retire early, sometimes even in a compelling manner (N=2). There was a threat of dismissal, or the employer caused a situation in which the employee felt it was not possible to continue working in an agreeable manner. This often occurred when organisations were restructuring. The push to early retirement by the employer was not directly caused by the health problems, but was related to the work situation that emerged due to these health problems. For example, a 61-year old man who worked for the largest part of his life for one company in various ICT-related jobs explained he retired early due to a complex interplay of “unwanted circumstances”. This man explained that work had always been very important to him. The largest part of his working life he worked 50 to 60 hours a week. Three years before he retired, he got a stroke. He recovered, but it took a year before he was able to start working again. His department had been sold to another company and his job did not exist anymore. He explained that he organized his own re-integration by arranging another job, and received little help from his employer:

“I have been out of circulation for a year. When I came back to [company name] there was no work anymore for me. I searched for a job within the organisation and found something.” […] “I organised my own reintegration. Nobody from the personnel department was concerned about that. It’s a pity, but it was just like that.”

He was overqualified for this job. The employer was not satisfied with this new situation. They made clear the company wanted to let him go, and proposed a financial arrangement for early retirement. This man was very disappointed in his company. Nevertheless, after tough negotiations, he accepted an adjusted financial arrangement. Although it was a choice to accept the proposed arrangement, it did not feel like a choice to him. The company at least did not create a situation in which this man could have decided to continue working in a pleasant way.

### Good health

‘Good health’ was (one of) the reason(s) to retire early in 5 of the 30 participants. This occurred also in persons who suffered from chronic diseases. These persons reported they wanted to enjoy life while their health still allowed them to do so. In most persons, the awareness of being in ‘good health’ arose, when they were confronted with the finiteness of life. This was caused for example by illness or death of a family member or friend, or death of a colleague immediately after retirement. A 62-year old administrative assistant expressed his fear to die or getting a handicap after years of hard working:

“Too often I’ve noticed this in entrepreneurs: they worked themselves half to death until they are at least 65, sometimes older… and when they retire they just die or they got sick, or they get a handicap and can’t do anything anymore. Well, I didn’t want that, I want to do things while I still can.”

This man also explained he wanted to spend more time with his wife:

“And because my wife didn’t work anymore and was also at home. And I said, listen, I’m healthy and there are things that I still want to do together.”

Persons were looking forward to having more time and freedom, and wanted to spend more time on hobbies, family and friends, and holidays. A 62-year old math teacher, who was very motivated to work, felt he had worked long enough and wanted to enjoy the time and the freedom of retirement now. About health he said the following:

“But you can also turn it around and say you’d be better off stopping while you’re still healthy so that you can still enjoy it.”

In these employees, continuing to work did not fit into their perspective of the future. What they wanted to do in life, while still in good health, did not match with what they expected to be able to do while working. These kind of processes occurred both in persons who enjoyed their work and found it important, and in persons who did not enjoy their work and merely did it for a living. Persons did not discuss opportunities to adjust certain job characteristics with their employer, such as working hours and flexibility, to reach a better fit between their job and private lives.

Although ‘good health’ was reported as a reason to retire early, it was often not a primary reason. Besides, it was always accompanied by the financial opportunity to retire early because of savings or a favorable early retirement scheme. One men, in which good health was an important reason to retire early, explained:

“And the fact that it was possible. Also financially.” […] “If it would not have been possible financially, I would have had to continue working.”

## Discussion

The aim of the present study was to identify through which pathways health influences early retirement. Face-to-face semi-structured interviews with thirty Dutch employees who retired before the age of 65 were conducted. Both poor and good health played a role in early retirement. Poor physical or mental health influenced early retirement through four different pathways, i.e. (a) persons felt unable to work at all due to health problems, (b) poor health resulted in a self-perceived decline in the (future) ability to work, (c) employees were afraid of a further decline in health, (d) or employees with a poor health felt pushed out by their employer, although they themselves did not experience a decline in their ability to work. Good health influenced early retirement, since persons wanted to enjoy life while their health still allowed to do so. The financial opportunity to retire sometimes triggered the influence of poor health on early retirement, and often triggered the influence of good health.

Earlier studies have shown that poor health is a predictor of early retirement [7]. However, it remained largely unclear how poor health influences early retirement. The qualitative nature of the present study allowed us to distinguish different pathways through which poor health influences early retirement. To the knowledge of the authors, the present study is the first study that was able to identify such pathways. Furthermore, the present study identified an additional health-related reason for early retirement, i.e. good health. This is in line with findings from van Solinge and Henkens [17], who showed that employees with a shorter subjective life expectancy more often intended to retire early than those who expected a longer life span. Our finding that good health, in addition to poor health, influenced early retirement, may partially explain why some previous quantitative studies did not find a significant relationship between health and early retirement [15].

In agreement with earlier studies, we found that the financial situation of the household and the opportunity to make use of various retirement schemes or financial arrangements played a role in early retirement [18]. The importance of financial factors differed between the different pathways. The financial opportunity to retire sometimes triggered the influence of poor health on early retirement, and often triggered the influence of good health on early retirement. At the time the interviews were conducted, early retirement schemes were highly accessible in the Netherlands. Leaving the labour market before the statutory retirement age was even described as an offer employees could not refuse [19]. Since these favorable arrangements will disappear in the near future, the financial opportunities to retire early will decline as well. This might affect the pathways via which health influences early retirement, and the proportion of employees in which it does so. For example, persons who experience they are not able to work due to ‘poor health’ may no longer retire early, but may receive disability pensions, or, if not eligible for this, may become unemployed. Persons who are afraid of a further decline in health may get more health problems. Furthermore, good health might still be a reason to retire early, but in a smaller proportion of the employees who can financially afford to retire early.

The relationship between health and early retirement seems to be in line with the person-job-fit approach [20]. This approach assumes that there needs to be a ‘fit’ between a person and his job to feel healthy, to enjoy work and to perform well in the job. Edwards distinguished two types of fit: demands-abilities fit and need-supply fit [21]. Demands-abilities fit exists if the demands of the job match with someone’s abilities. Need-supply fit exists if attitude and motivation of an employee match with the work context. Accordingly, in the present study poor health influenced early retirement due to a misfit between the job demands and the (perceived future) ability to perform the working tasks, without worsening health problems. Good health influenced early retirement due to a misfit between what people wanted to do in their lives while being in good health, and what they expected to be able to do while working.

Employers and employees barely discussed the ‘person-job-misfit’. This could be illustrated by the fact that employees who retired early due to a self-perceived (future) decline in their ability to work or fear of a further decline in health, did not discuss these perceptions or anxieties with their employer. Similarly, employers who pushed employees with poor health out of work did not discuss their plans from an earlier phase onwards. A dialogue between employees and employers and measures directed to the prevention of (future) misfits might be helpful to prolong working life in good health and with good work ability. The different pathways by which health influences early retirement suggest that these measures need to be tailored to the individual. For example, it could be hypothesized that the 63-year old job coach, who retired early since he experienced a decline in his ability to work, could have continued working if he had received other responsibilities than “to pump people up”. Besides, it could be hypothesized that the 62-year old math teacher might have continued working if flexible working hours would have enabled him to enjoy the things he liked outside of work. We recommend future research to investigate why this dialogue between employers and employees is often lacking and what an effective dialogue should include.

A major strength of the present study is the qualitative design, which allowed us to explore *how* health influences early retirement. Another strength of the present study is that we conducted interviews with persons who had retired early, or already formally arranged to do so within six months after the interview, instead of persons who only intended to retire early. Hence, we studied early retirement behavior. Previous studies often investigated the intention to retire early [7,22,23] or continue working [24], but factors that influence the intention to retire early may differ from those that influence actual early retirement [17].

However, this study also has limitations. First, in qualitative studies the researcher is an important instrument, both in data collection and data analysis [25]. This may have influenced our findings. Therefore, analysis of the interviews was predominantly done by two persons. Moreover, to enhance robustness of the findings, main results were discussed with other co-authors as well. Second, during the interviews, persons looked back at their transition from work to early retirement. There is a risk of recall-bias and to transformation of the ‘real’ story, since persons may not remember facts correctly or may be influenced by psychological processes, such as cognitive dissonance. The interviewer used in-depth follow-up questions to validate the answers of the interviewees. Moreover, to prevent bias of our results we checked for inconsistencies in the stories and interpreted these parts with caution. Third, we only studied the role of health in early retirement in persons who retired early. Hence, we have no insight in the role of health among persons who had the opportunity to retire early, but continued working. To further establish the role of health, we recommend future research to investigate how health influences the prolongation of working life. Furthermore, it should be noted that we only found evidence of five different pathways from health to early retirement in the present study. Future studies should replicate this finding and may reveal additional pathways. Finally, the present study focused on the influence of health on early retirement. It is important to keep in mind that other factors (e.g. financial situation) play an important role in early retirement as well.

## Conclusions

Taken together, poor and good health influence early retirement via several different pathways. To prolong working life, a dialogue between employers and employees, from an early phase in the career onwards, and work-related tailored interventions may be helpful.

## Competing interests

The authors declare that they have no competing interests.

## Authors’ contributions

All authors made a substantial contribution in the design of the present study. AdW and KR were responsible for data collection. AdW and KR analyzed the interviews and AdW, GG, KR, and MW regularly discussed the findings. All authors read and approved the final manuscript.

## Pre-publication history

The pre-publication history for this paper can be accessed here:

http://www.biomedcentral.com/1471-2458/13/292/prepub
